# Psychosocial and Sociodemographic Determinants Related to Chronic Diseases in Immigrants Residing in Spain

**DOI:** 10.3390/ijerph19073900

**Published:** 2022-03-25

**Authors:** María José Martos-Méndez, Luis Gómez-Jacinto, Isabel Hombrados-Mendieta, Anabel Melguizo-Garín, Iván Ruiz-Rodríguez

**Affiliations:** 1 Faculty of Psychology, University of Málaga, 29071 Málaga, Spain; mihombrados@uma.es (I.H.-M.); anamel@uma.es (A.M.-G.); ivan_dlr@uma.es (I.R.-R.); 2 Faculty of Social and Labor Studies, University of Málaga, 29071 Málaga, Spain; jacinto@uma.es

**Keywords:** chronic diseases, social determinants, sociodemographic factors, health, immigrants

## Abstract

The aim of the study is to analyze the effect of the psychosocial determinants of satisfaction with social support, resilience and satisfaction with life, and the sociodemographic determinants of age, gender and length of residence on chronic diseases in immigrants living in Spain. The sample was composed of 1131 immigrants from Africa, Eastern Europe, Latin America and Asia. 47.1% were men and 52.9% were women. Most relevant results point to age as the sociodemographic variable with the highest predictive effect in the six chronic diseases analyzed. Gender, in this case female, predicts arthrosis, chronic back pain and migraine, whereas length of residence was only significant in the case of chronic allergies. Regarding psychosocial variables, resilience is a good predictor of hypertension, chronic allergies and arthrosis. However, satisfaction with social support appears to be the best predictor for chronic back pain in the regression equation, satisfaction with life being a significant variable in migraine, arthrosis, allergies and high cholesterol. Results are notably relevant for the design of preventive health programs in immigrants, as well as in ensuring their appropriate access to the health system so that their chronic diseases can be diagnosed. Given the relevance and incidence of the chronic diseases analyzed in immigrants, preventive strategies should be improved to tackle chronic diseases that can have a serious impact on immigrants’ health.

## 1. Introduction

### 1.1. Health and Migration

Immigrants’ health has been analyzed in recent studies [1,2,3,4]. According to the World Health Organization (WHO) (2019), health problems in immigrants are similar to those of the general population, although there is higher prevalence among certain groups and countries of origin [5]. Some studies show conclusive results on immigrants’ lower health compared to native populations [6]. Particularly, immigrants suffer from headaches and symptoms of burnout, which are interpreted as physical consequences of cognitive overload due to the migration process [7]. Cumulative exposure to chronic stress has been seen to contribute to the worsening of health in immigrants who have lived in host countries for longer periods of time [8,9]. This means that migrating can have a negative impact on individuals’ health due to multiple physical and psychosocial tensions suffered during the migration process [10,11]. These tensions can lead to high levels of stress and an increase in risk-behaviors in immigrants, thus affecting their overall health [12,13,14,15] and potentially causing them to develop certain chronic diseases or worsening existing conditions.

The long-term negative consequences of chronic diseases on health are widely known. Studies such as the one carried out by Agyemang et al. (2021) suggest that levels of type 2 diabetes are higher among immigrant populations in Europe compared to those of native populations [10]. Causes can be related to pre-migration factors as well as factors related to the migration process. A pioneering study carried out by Fakhoury et al. (2021) analyzed health perception in both undocumented immigrants and recently arrived documented immigrants in Geneva (Switzerland) [16]. Documented immigrants who had recently arrived in the country reported higher levels of perceived health, although a strong relation between social support and health perception was found in both groups. These results suggest the importance of social determinants involved in immigrants’ health. Ponce-Blandón et al. (2021) studied the consequences of migration in the Strait of Gibraltar and concluded that it was necessary to attend to the personal and social factors of immigrants in order to provide them with quality healthcare [17]. Poteat et al. (2021) studied stress models in minority groups within the Hispanic population in the United States and a relationship with the risk of cardiovascular disease was found [12]. This increase was mediated by social support and coping mechanisms, which would act as resilience factors against stress in the dimension of disease risk. Suglia et al. (2021) observed a higher prevalence of cardiovascular risk in Hispanic/Latino and African American groups, as well as higher rates of poverty and social stress compared to the non-Hispanic population in the US [18], thus highlighting the importance of considering social factors involved in these relations. A systematic review by Salami et al. (2021) analyzed migration within the African continent and found that minor African immigrants and refugees experience higher health problems linked to social factors such as lack of social inclusion or access to health care services [19]. These studies conclude, in general terms, that it is necessary to identify which psychosocial and sociodemographic factors can have an impact on immigrants’ health in order to improve programs and healthcare provided to immigrants by institutions. At present, there are few studies that analyze the influence of sociodemographic and psychosocial variables on chronic diseases in immigrants, both worldwide and in Spain. This is due to a lack of research grouping all variables in the same study based on specific chronic diseases. The present study focuses on Spain due to several reasons, such as it being a country with high immigration rates with people of various origins [20]. A heterogenous sample such as the one found in Spain can be of relevant interest. The aim is, therefore, to study some of the most common health problems experienced by immigrants in Spain and to identity which variables are the most closely related to certain chronic diseases.

Migration must be understood as a process during which an individual faces a series of events that are overcome in different stages [21]. Some of the multiple stressors which immigrants face include adapting to a new culture and social norms, language barriers, working in precarious conditions, economic difficulties, problems to regularize their legal situation in the host countries and the loss of social support networks [21,22,23,24]. Due to these stressors, there can be a higher incidence of chronic diseases in immigrants than in the native population, as observed in the previously mentioned studies. Furthermore, once such diseases are diagnosed, immigrants seem to manage these conditions more poorly than the native population, as they seem to adhere less to the corresponding treatments [25].

### 1.2. Psychosocial Factors and Chronic Diseases

Social relations have a positive impact on immigrants’ health, and factors such as family relations, social support from relatives and friends, and cohesion with the neighborhood or the community are indicators of optimal levels of health [26]. The protective nature of social support networks has been seen in multiple studies, as it can also be seen in the recent revision by Garcini et al. (2016) [22]. These studies show the protective nature of social support networks in the development of diseases in immigrant populations [14,27,28]. Social support can act as a coping agent and a mediating variable between stress due to acculturation suffered by immigrants and their health [15,29,30]. Shared experiences related to the migration process, social support, social inclusion and socio-economic factors play a key role in the appearance of certain chronic diseases [31,32,33].

Research carried out on immigrants living in Spain confirms better health in those immigrants with strong support networks and high satisfaction with support received [13]. In essence, those negative effects on immigrants’ health can be mediated by each individual’s resources and the context, for example, social support [15], as well as resilience, which is a highly important personal resource that can have a buffering effect over the negative effects of migratory stress. Resilience seems to be a personal resource that helps one cope positively with adverse situations and represents the added ability of knowing how to overcome certain adversities, learn from them and become stronger due to the experience [34]. Immigrants with high resilience might therefore be able to cope with the process of migrating and the stress caused by it in a more positive manner, making resilience a protective factor of immigrants’ health. Some studies have also found a relation between resilience and immigrants’ better adjustment in host countries [35], which could mean that higher social inclusion can lead to higher resilience and better adaptation in the host community.

There are few studies that analyze immigrants’ resilience. Gutierrez and Telmo (2006), in a study on resilience in Peruvian immigrant women [36], concluded that different levels of resilience were determined by a group of variables such as personal characteristics, family history, support networks, cultural identity, adaptation methods, acquisition patterns of Spanish culture and, particularly, the length of time since they arrived in the host country. Pereda (2006) also pointed to some risk factors for resilience in the migration context [37] organized in three dimensions: the individual level (i.e., lack of coverage in basic needs), the family level (i.e., family re-grouping problems), and the social level (i.e., isolation and lack of support). In the same line, he also suggested that social support is related to immigrants’ resilience, linking it to less probability of suffering depression and the lower risk of hospital admissions [38,39]. Resilience’s relation with adversity is what makes it a variable of interest to be studied in immigrant populations, since, as it has been suggested by some authors, resilient individuals are more able to integrate in host communities and develop a sense of community, which is particularly important for minorities and immigrant populations [40,41,42].

Difficulties derived from the migrating process can also affect satisfaction with life and immigrants’ general well-being [43]. However, there are some studies that do not confirm the existence of a harmful and direct effect of migration on immigrants’ well-being [44]. What several studies do confirm is the different situations that every immigrant goes through to some extent [45,46,47]. These include, for example, loss derived from leaving the country of origin, frustration, and cultural shock due to cultural differences, legal, residential and socio-economic difficulties in the host country, and the rejection and discrimination by some individuals and/or groups [48]. All these changes can affect immigrants’ quality and satisfaction with life. Several studies confirm a relationship between social support and satisfaction with life in immigrants, meaning that the relations immigrants have with their neighbors and community members have a very positive impact on their health, well-being and satisfaction with life [49]. Likewise, having support for information and resources from religious, political and social associations has also been observed to improve immigrants’ well-being [50,51,52].

### 1.3. Sociodemographic Variables and Chronic Diseases

Sociodemographic characteristics of immigrants have been widely studied, since different social circumstances affect immigrants’ well-being and health differently. Some of the most relevant characteristics include age, origin, gender, education level, civil status, number of children, length of residence, social and work situation, reason for migrating, legal status and language command. Dominguez and Hombrados (2007) analyzed sociodemographic characteristics of immigrant women [53], and found that a high percentage of them had migrated alone, without family support and for economic reasons. They also found that despite those women having medium-high education levels, their work activity was almost exclusively limited to domestic service.

In numerous studies, sociodemographic variables appear to predict satisfaction with life and health. Most satisfied individuals are those who are married or live with their partners, those who have lived in the same place for several years and those with higher education levels [54]. However, very often these characteristics appear differently in immigrants since they are usually far from their partners and family, have lived for less time in the same place and have lower economic status. It is also widely known that age and country of origin can correlate with some health problems and predict health in immigrants.

Spain, the country where the study was carried out, has a high percentage of people over 65 (19.95%), meaning it has an ageing population. Spain also has a considerably low birth rate, only 7.19%, and the percentage of immigrants is high, almost 12% [20]. There are also high unemployment rates throughout the country. In the case of immigrants, they might be more vulnerable to unemployment due to being new to the country and having less resources for their employment search, thus leading to higher instability which in turn affects their health. This generates economic inequalities for those immigrants who cannot access employment. The COVID-19 pandemic has affected immigrants’ economic instability to a higher degree than the native population [55]. Spain has a female population of 50.1% [20], but there is still a considerable gender gap. Women have higher unemployment rates than men. Therefore immigrant women experience higher instability in almost every sense, including economic instability, which is related to poorer health and quality of life [53].

### 1.4. Present Study

Research on immigrants’ health tends to analyze health from a general perspective, without considering specific symptoms or diseases. Therefore, the present study aims to identify which psychosocial and sociodemographic variables have stronger predictive effect on certain chronic diseases in immigrants.

This approach on diseases aims at obtaining necessary information to know the characteristics of health problems experienced by immigrants, and ultimately identify which strategies can be designed for psychosocial intervention to improve immigrants’ health and quality of life. In the model suggested, all sociodemographic and psychosocial variables appear as antecedent variables or variables with predictive value for the specific health problems or chronic diseases analyzed.

From a psychosocial point of view, and based on previous research [10,12,16,18], the present study aims to analyze the sociodemographic and psychosocial factors that affect chronic diseases in immigrants. For such purpose, common chronic diseases in immigrants have been selected based on long-term effects that can be considered as high risk for immigrants’ health.

Psychosocial variables analyzed are those with higher relation to immigrants’ health, such as social support, resilience and satisfaction with life [21,22,23,24]. In the case of sociodemographic variables, we chose those with high relation with certain health symptoms or problems, that is, age, gender and length of residence in the host country [53].

The following hypotheses are derived from the objectives of the study:

- Sociodemographic factors will have an impact on health problems. More specifically, higher age, longer residence time and being a woman will relate to increased chronic disease.
- Satisfaction with social support, resilience and satisfaction with life relate with health. Lower levels of social support, resilience and satisfaction with life will have a negative impact on immigrants’ health.

## 2. Materials and Methods

### 2.1. Participants

The sample included 1131 immigrants from Africa (24.2%), Eastern Europe (23.7%), Latin America (27.4%) and Asia (24.7). The distribution of immigrants in the city represents data from the 2019 census. 47.1% were men and 52.9% were women. The average age was 32.26 (d.t. = 11.84) and the average length of time participants had been living in Malaga (Spain) was 10.35 years (SD = 7.30). Most immigrants had left their countries of origin with the purpose of improving their financial situation (48.8%); secondly, they had left with their families (26.9%), and the remaining percentage represents those who had left their countries of origin due to the political situation, in order to study or due to their sexual orientation or identity. These figures represent the distribution of immigrants in Malaga according to data from the 2018 census. In accordance with the Council of Malaga (2018) [56], on 1 January 2018, the number of immigrants in Malaga was 573,832, which is 6.04%. A sample was determined for this population, following a formula for finite universes with a reliability level of 99% and a margin of error of ±5. Regarding the relative weight of the different origins based on the presence of each continent in Malaga, Ukraine and Romania are to be noted (26.68%) from Europe; Morocco (69.39%) and Nigeria (17.35%) from Africa; Paraguay (28.18%) and Argentina (13.44%) from the Americas; and China (74.80%) from Asia.

### 2.2. Procedure

Participants were selected using sampling methodology and random route. Limits were set for each neighborhood selected and random route sampling was used to designate the buildings, streets, sidewalks, etc. in each area. Interviewers were duly trained and oversaw the handing and administering of questionnaires to participants. Questionnaires were collected in the city of Malaga from the 11 districts of the city and the 11 towns of the region of Malaga with higher numbers of immigrants. Questionnaires handed to non-Spanish speakers had been duly translated into the respective languages by bilingual native speakers who had a perfect command of Spanish. For both languages to match, translators read the questions and ensured participants had understood the objective of each section. Answers were subsequently gathered by interviewers according to the methodology suggested by the transcultural research of Páez and Vergara (2020) [57]. Questionnaires were handed out at immigrant community centers, businesses, meeting points and social service centers located in each district. All immigrants who participated did so voluntarily and signed informed consent forms. No incentives were offered for their participation. The Ethical Committee of the University of Malaga (CEUMA: 37-2016-H) approved the fitness of the study’s protocol, the informed consent, the professional conduct, the experimental procedure and the ethical criteria. The instrument used to gather data used for the questionnaire was developed by grouping under a single document the questionnaires on diseases, social support, satisfaction with life, resilience and sociodemographic variables. This instrument for data collection was applied in a single meeting with each participants and the duration ranged between 30 and 40 min.

### 2.3. Measures

A questionnaire including all the instruments used to analyze the study’s variables was designed. Those instruments are the following:

Illness. Spanish Statistical Office (2019) [20]. This questionnaire comprises a list of 28 illnesses or health problems. The present study only included items that were related to hypertension, migraine, arthrosis, chronic back pain, high cholesterol and chronic allergy. Participants were asked questions related to whether they had experienced some of those problems over the past 12 months (“Have you experienced this problem over the past 12 months?” 1 = Yes, 2 = No).

Satisfaction with life. The five-item Satisfaction with Life Scale (SWLS) [58], developed by Pavot and Diener (1993) was used to assess life satisfaction or the cognitive component of well-being. Items were answered on a 7-point scale ranging from 1 = completely unsatisfied to 7 = completely satisfied (i.e., “I am satisfied with my life”). This scale has a Cronbach’s α = 0.88. Diener showed an Alpha coefficient of 0.87 for the scale and a stability coefficient of 0.82 after re-testing two months later. These features are particularly relevant when the scale is applied to detect changes in satisfaction with life in populations that have experienced big changes in their lives, such as immigrants.

Resilience. The reduced version of 10 items of the CD-RISC Scale of Resilience by Connor and Davidson (2003) was used [59]. This scale uses a Likert-type format ranging from 1 = completely agree to 5 = completely disagree. Some items include statements such as: “I can overcome anything” or “I try to see the positive or fun side when I face a problem”. Cronbach’s Alpha showed a value of 0.93.

Social support. The Questionnaire of Frequency and Satisfaction with Social Support by García, Hombrados-Mendieta and Gómez (2016) [60] measures both the frequency of support received and the satisfaction experienced by the subject in relation to such support. Its design allows for the measuring of these two aspects based on the three most studied types of support in the literature (emotional, instrumental and informational) and the different sources of social support. The sources of support analyzed in the present study are family, immigrant friends, local friends, neighbors and community members. Sources, type, frequency and satisfaction with support in each individual can be measured with this instrument. In the present study, a general measure of satisfaction with support including all the types and sources was used. The level of satisfaction with support received is measured with a 5-option Likert scale (1 = Very dissatisfied; 2 = Very little satisfied; 3 = More or less satisfied; 4 = Quite satisfied and 5 = Very satisfied). This instrument has been previously used with immigrants and it has shown high internal consistency. Cronbach’s alpha was 0.80 for both the scales of frequency and satisfaction with support.

Sociodemographic characteristics. A questionnaire was designed including questions on the main sociodemographic characteristics of participants, such as nationality, age, gender, civil status, length of residence in the host country, etc.

### 2.4. Data Analysis

Data was analyzed with the statistic program IBM SPSS version 23 (IBM, Armonk, NY, USA). Descriptive analyses were performed to describe sociodemographic, psychosocial and health variables. The study takes a close look at those chronic diseases that appear to be most common in the sample of participants analyzed. In order to link psychosocial and demographic variables to chronic diseases, the usual procedures used in medicine to predict such relations were used. A binary logistic regression analysis was carried out on some of the diseases that immigrants expressed having felt over the past year. All cases included dichotomous variables, that is, having them or not having them. Psychosocial variables such as resilience, social support and satisfaction with life, and the demographic variables of age, gender and length of residence were included as predicting variables in the binary logistic regression analyses. Dependent variables were analyzed for each health condition.

## 3. Results

Table 1 shows the frequency and percentage of each disease by gender. Table 2 shows descriptive results of the sociodemographic and psychosocial variables. Regression analyses were carried out based on the N values shown in Table 2, which correspond to each variable analyzed.

Table 3 shows the results on hypertension and migraine. Age and resilience are the variables that better explain hypertension. Values from the chi-square test show that these two independent variables explain arterial hypertension. As it can be observed, the R^2^ ranges between Cox and Snell’s R-square and Nagelkerke’s R-square. This means that both variables explain up to 12.1% of the variance of arterial hypertension. The correctly classified global percentage, which indicates the number of cases the model is able to predict correctly, is high, above 50%. Results show that higher age relates to higher hypertension and higher resilience relates negatively to hypertension. Results related to migraine or chronic headache, gender and satisfaction with life are those that better explain this condition. As it can be observed, R^2^ ranges between Cox and Snell’s R-square and Nagelkerke’s R-square, meaning that being female gender and satisfaction with life explain up to 5.4% of the variance of migraine. The correctly classified global percentage, which indicates the number of cases the model is able to predict correctly is 75.5%. Results show that an increase in satisfaction with life relates to a decrease in migraine and that women suffer it more (62.8%) than men (37.2%).

Table 4 shows results on arthrosis and chronic back pain. Four variables have higher explanatory capacity for arthrosis: resilience, satisfaction with life, gender and age. Chi-square values indicate that these four independent variables explain arthrosis and back pain. As it can be observed, the R^2^ shows that these four variables explain up to 14.3% of the variance of arthrosis. The correctly classified global percentage is 95.0%. Results show that lower satisfaction with life relates to higher arthrosis, and higher resilience relates to higher arthrosis. They also show that higher age relates to higher arthrosis and that being a woman relates to the higher probability of suffering arthrosis as well. Global indexes for chronic back pain are also good, with age, gender and satisfaction with social support in the equation. Age has a positive and statistically significant relation with having suffered from chronic back pain. On the contrary, social support relates negatively with this condition, thus indicating that higher social support relates to lower chronic back pain. Additionally, regression analyses show that female gender better predicts this condition than male gender does. The R^2^ ranges between Cox and Snell’s R-square and Nagelkerke’s R-square, meaning that the three variables explain up to 4.7% of the variance of chronic back pain. The correctly classified global percentage is 81.6%.

Table 5 shows the results regarding high cholesterol and chronic allergy. Global indexes on cholesterol are good, with satisfaction with life and age included in the equation. Higher age relates to an increase of suffering high cholesterol, and lower satisfaction with life shows higher cholesterol as well. R2 indicates that these two variables explain up to 15.3% of the variance of cholesterol. The correctly classified global percentage is 90.7%. Global results on chronic allergy seem similar to the ones on cholesterol and they indicate a good adjustment of the model. Age, length of residence, resilience and satisfaction with life were included in the equation. Age and satisfaction with life show a negatively and statistically significant relation with having suffered allergy problems. Length of residence and resilience also relate positively and significantly to allergy. However, the four variables only explain 2.6% of the variance of chronic allergy. The correctly classified global percentage is 86.5%.

## 4. Discussion

The aim of the present research is to identify which sociodemographic and psychosocial variables can affect certain chronic health conditions in immigrants in Spain. Results have shed light on the matter and show which variables might have a certain predictive level on some of the chronic diseases analyzed.

Social status, gender, ethnical origin or place of residence are some of the social determinants that explain the distribution of health in immigrant populations to a high extent [11,61,62,63,64]. Based on the results obtained, age is the variable with the highest relation with the chronic diseases analyzed. This means that many health conditions are tightly related to individuals’ age. Results show that hypertension, arthrosis, chronic back pain, cholesterol and chronic allergy relate to age, which is in line with other studies [65]. Since age is a controlled variable in the regression analyses carried out, these results are notably significant. They mean that the rest of the variables which influence some of the health conditions analyzed have an even higher relevance, since age is not influencing those relations.

Gender is also observed to be a predictor of some health conditions. As observed in other research [11,66], gender is notably relevant as a health determinant in immigrant populations, with women suffering more health problems [67], particularly arthrosis, back pain and migraine. These results are in line with those obtained by Zou et al. (2021), who performed a systematic review on immigrant women and physical activity [68]. Immigrant women engage less in physical activities due to barriers related to social determinants, which can in turn have a negative impact on their health. Results found in the present study are relevant for the design of prevention and health promotion programs in immigrants, since intervention strategies, in the case of immigrant women, should address those diseases that affect them more than men. In this sense, we suggest designing gender-based intervention programs which identify the needs and suggest specific goals for immigrant women.

The length of residence in the host country relates to chronic allergy in the sense that the higher the length of residence the higher the probability of suffering this condition. In this sense, some research has found that immigrants, once they settle in the host country, adopt the habits of the native population, which in many cases can be harmful. One example of this can be the diet and nutrition, which is different for immigrants compared to the diet they followed in their countries of origin, and which can lead to serious health problems, some of them related to allergies [4,69] or more serious conditions. Those immigrants who have lived for a longer time in the host country are no longer bound to such positive selection in terms of health, as defended by the theory of “the healthy immigrant effect” [70,71] and can thus increase the vulnerability of suffering different diseases and modify behavioral and lifestyle patterns [72,73]. Additionally, cumulative exposition to chronic stress has been observed to worsen the health of those immigrants who have lived a longer time in the host country [8,9], potentially harming their health, for example in terms of developing chronic diseases. Other studies such as the one conducted by Buchcik et al. (2021) found that the level of education related to mental health and stress in immigrants in Hamburg (Germany) [74], meaning that along with length of residence, the level of education seems to also be related to immigrants’ health [75] as well. In the same line, the research by Toselli et al. (2018), which looked at sociodemographic and psychosocial variables in immigrants in Italy, concluded that education and the number of children were related to immigrants’ health and quality of life [76]. This study also analyzed the relationship between anthropometric and quality of life variables of immigrants. It would be of interest to explore this line in future research to study the relation of these variables with chronic diseases in immigrants.

In essence, the three sociodemographic variables analyzed in the study have a certain predictive effect on some health determinants, thus confirming the first hypothesis of the study. Age was the variable with the highest influence on the health conditions analyzed.

Satisfaction with life, social support and resilience showed a significant relation with some of the health conditions analyzed, therefore also confirming the second hypothesis of the study.

Satisfaction with social support proved to be a significant variable in the explanation of chronic back pain. Data obtained in other research confirm that some of these health conditions are more notable and probable among those individuals who lack social support, or the social support they receive is insufficient or unsatisfactory [77,78]. Deficient levels of social support relate to the risk of cardiovascular diseases [12,18,79], psychological problems [80,81,82], or low levels of health [16,83]. In this sense, interpersonal relationships can act as protective factors against different physical and mental problems. Therefore, social support would play a relevant role as a moderating variable between stress by acculturation and symptoms of anxiety and depression in immigrants [84,85]. Likewise, the perception of social support relates positively to coping mechanisms that reduce stress and health problems [12,86]. Studies such as the one carried out by Jia et al. (2022) analyze coping mechanisms among other factors and how these affect immigrants’ health [11].

On the other hand, results from the present study show a negative and significant relation between resilience and hypertension. Immigrants’ ability to overcome the crises derived from the migration process and the adversities related to them, knowing how to recover and, above all, overcoming them successfully by growing personally has a significantly positive impact on their health. More resilient immigrants would be therefore less affected by physical and mental problems or would at least face them more successfully. Likewise, social support relates to immigrants’ resilience ability, and it is linked to a lower probability of depression and hospital admission [12,38,39]. Other studies such as the one carried out by Kong et al. (2021) also studied the relationship between social support received and resilience on immigrants’ health including the variable of self-efficacy, which moderated resilience and social support [87]. A positive relation was also found between arthrosis and chronic allergy and resilience, which interestingly differs from previous studies. A possible explanation for this is that, as indicated by other studies, resilience relates to immigrants’ higher adjustment and inclusion in the host country [37], and that due to such immersion in the host country, immigrants might adopt certain social habits which can in fact be harmful. However, resilience has been little studied in immigrants and even less so its relation to specific health problems, making the present preliminary results important as a starting point of reference which suggests the need to further analyze the relation between resilient factors and immigrants’ physical and mental health.

The relation between satisfaction with life and health has been confirmed in a wide number of studies [80,88,89]. It is important that individuals feel healthy both in terms of health conditions as well as satisfied with their life. Sometimes, the latter is even more important than not experiencing physical pain [90]. Results show that satisfaction with life has a certain predictive value on some health conditions such as arthrosis, migraine, allergy and cholesterol—those immigrants who feel less satisfied with life express suffering from these conditions more often. You et al. (2021) analyzed the psychosocial factors related to the health of Chinese immigrants, and in this case, among other variables, they studied the subjective social status of immigrants in relation to satisfaction with life and health [75]. In fact, and since health is not limited to the absence of diseases but is rather an overall feeling of physical, mental and social well-being, it seems reasonable that those individuals who feel more satisfied with their lives also feel healthier. Additionally, the psychosocial approach to health understands the processes of health/illness as dependent on transactions between the individual and their environment [91]. This means that the social context, the culture, and interpersonal relationships are relevant to explain the health problems of a certain individual, collective or community, which is notably relevant in the case of immigrants.

Finally, and according to the results from the study, it is necessary that strategies and action plans be developed at the European and Spanish level to improve immigrants’ health by creating new health policies adapted to their needs. The Spanish national health system helps protect the health of most of the population, including immigrants. However, there is room for improvement, and new intervention programs should be designed with the purpose of promoting good health practices among diverse communities [92]. For instance, it would be necessary to provide better quality and accessible health coverage as well as social protection for all immigrants regardless of their legal status, so health systems can become more respectful of cultural and linguistic differences [5]. Likewise, immigrants with chronic health conditions should receive follow-up and control. Many immigrants remain undiagnosed due to not having easy access to the health system, which increase the risk of suffering from an undiagnosed chronic disease. Therefore, health strategies and policies that address the immigrant population with chronic health conditions are very much necessary.

### 4.1. Limitations of the Study

Among the limitations of the study, its cross-sectional design is to be noted. It would be convenient to carry out a longitudinal study to obtain more information on relations between variables and how these affect each other in the long term. Additionally, the study was done following a quantitative methodology, which thus limited it. Wider research could be carried out by applying a more qualitative analysis. The immigrants’ city of residence could also be considered as a limitation, since all participants live in Málaga (Spain). Future lines of research will include data gathered from other locations in Spain. The high number of participants and the gender balance is a strength of the study. Likewise, the different countries and areas of origin of immigrants are well represented, thus leading to the high representation of ethnical origins. Finally, the study includes chronic health problems and the analysis of both sociodemographic and psychosocial variables that affect such health conditions. This means that the study goes further than the general analysis of health on immigrant populations, delving into specific health conditions with high incidence in this collective.

### 4.2. Future Lines of Research

Other variables which can be of interest to improve the knowledge on social determinants affecting immigrants will be considered in future lines of research. Knowing immigrants’ stress levels, their sense of community or their satisfaction with the social support they provide to their networks (not only satisfaction with social support received) can be variables that affect immigrants’ health. It could also be of interest to know what other sociodemographic variables affect immigrants’ health, such as the number of children they have, the number of people under their care, income, education, and occupation, etc. Considering social determinants of health based on gender could also be an interesting future line of research; when speaking of care, we must not forget that women are usually the ones who provide it compared to men [93], and unemployment and economic inequality are higher amongst female immigrants, and thus can have an impact on their health. Finally, it is also important to consider the impact of the COVID-19 crisis on immigrants’ health and how this situation might be mediating or promoting to some extent the social determinants and psychosocial risk factors that can have an impact on their health.

## 5. Conclusions

Based on the results from the present study, it can be concluded that considering psychosocial and sociodemographic factors in immigrant populations in Spain is necessary for the design of social action plans, but also to provide health services that are more adapted to this collective’s specific needs. As was mentioned in the previous section, the current crisis caused by COVID-19 has stressed the great difficulties most vulnerable populations face. In particular, immigrants have seen their psychosocial situations worsened, thus affecting their health [80,81,82,83,84]. Being an immigrant and suffering from a chronic disease such as high cholesterol, diabetes or hypertension can be a risk factor that can lead to other diseases such as COVID-19 [94]. Social support and the promotion of support networks can buffer immigrants’ current situations with regard to their difficulty accessing health services [80,94]. In the current context of health systems encompassed by the COVID-19 sanitary crisis, it is more necessary than ever to attend to the risk factors and social determinants of health in general and immigrant populations in particular, since giving this matter the attention it requires can contribute to improved quality of life and the perception of health.

## Figures and Tables

**Table 1:** Chronic diseases by gender (*N* = 1131).

	Gender					
	Women		Men		Total	
	Cases	%	Cases	%	Cases	%
Hypertension	73	45.6%	87	54.4%	160	100.0%
Arthrosis	38	62.3%	23	37.7%	61	100.0%
Chronic back pain	125	59.2%	86	40.8%	211	100.0%
Cholesterol	51	50.5%	50	49.5%	101	100.0%
Migraine	177	62.8%	105	37.2%	282	100.0%
Chronic allergy	79	52.0%	73	48.0%	152	100.0%
No disease reported	56	34.2%	108	65.8%	164	100.0%

**Table 2:** Descriptive statistics of psychosocial and sociodemographic variables.

	Mean	Typical Deviation	*N*
Age	32.26	11.8	1091
Time of residence	10.30	7.11	1097
Satisfaction with social support	3.83	0.81	1073
Resilience	3.79	0.60	1118
Satisfaction with life	4.42	1.24	1113

**Table 3:** Logistic regression analysis of hypertension and migraine.

Hypertension	B	E.T.	Wald	Gl	Sig.	Exp(B)
Age	0.061	0.008	54.416	1	0.000	1.063
Gender(1)	−0.188	0.188	1.004	1	0.316	0.829
Length of residence	−0.017	0.013	1.691	1	0.193	0.983
Satisfaction with social support	0.150	0.129	1.351	1	0.245	1.162
Resilience	−0.357	0.182	3.845	1	0.050	0.700
Satisfaction with life	−0.084	0.088	0.922	1	0.337	0.919
Constant	−2.452	0.677	13.112	1	0.000	0.086
R^2^ = 0.069–0.121/χ^2^ = 70.407 (gl = 6, *p* < 0.001)/% global = 85.7						
**Migraine**	**B**	**E.T.**	**Wald**	**Gl**	**Sig.**	**Exp(B)**
Age	−0.004	0.07	0.38	1	0.537	0.996
Gender(1)	0.744	0.157	22.517	1	0	2.105
Length of residence	−0.012	0.012	1.023	1	0.312	0.988
Satisfaction with social support	−0.128	0.104	1.525	1	0.217	0.88
Resilience	0.162	0.151	1.15	1	0.284	1.175
Satisfaction with life	−0.223	0.072	9.677	1	0.002	0.8
Constant	−0.429	0.564	0.578	1	0.447	0.651
R^2^ = 0.036–0.054/χ^2^ = 33.463 (gl = 6, *p* < 0.001)/% global = 75.5						

**Table 4:** Logistic regression analysis of arthrosis and back pain.

Arthrosis	B	E.T.	Wald	Gl	Sig.	Exp(B)
Age	0.062	0.013	23.722	1	0.000	1.064
Gender(1)	0.654	0.322	4.125	1	0.042	1.922
Length of residence	−0.010	0.020	0.247	1	0.619	0.990
Satisfaction with social support	−0.146	0.203	0.514	1	0.474	0.865
Resilience	0.918	0.315	8.503	1	0.004	2.504
Satisfaction with life	−0.405	0.140	8.371	1	0.004	0.667
Constant	−6.824	1.223	31.132	1	0.000	0.001
R^2^ = 0.046–0.143/χ^2^ = 46.615 (gl = 6, *p* < 0.001)/% global = 95.0						
**Chronic Back Pain**	**B**	**E.T.**	**Wald**	**Gl**	**Sig.**	**Exp(B)**
Age	0.025	0.007	11.141	1	0.001	1.025
Gender(1)	0.358	0.17	4.398	1	0.036	1.43
Length of residence	0.009	0.012	0.52	1	0.471	1.009
Satisfaction with social support	−0.220	0.113	3.775	1	0.05	0.802
Resilience	0.213	0.169	1.586	1	0.208	1.237
Satisfaction with life	−0.088	0.079	1.237	1	0.266	0.915
Constant	−2.203	0.627	12.338	1	0	0.11
R^2^ = 0.029–0.047/χ^2^ = 28.958 (gl = 6, *p* < 0.001)/% global = 81.6						

**Table 5:** Logistic regression analysis on cholesterol and chronic allergy.

Cholesterol	B	E.T.	Wald	Gl	Sig.	Exp(B)
Age	0.076	0.010	57.431	1	0.000	1.079
Gender(1)	−0.008	0.233	0.001	1	0.974	0.992
Length of residence	−0.028	0.016	3.137	1	0.077	0.972
Satisfaction with social support	0.242	0.159	2.312	1	0.128	1.274
Resilience	−0.129	0.224	0.333	1	0.564	0.879
Satisfaction with life	−0.214	0.107	3.963	1	0.047	0.808
Constant	−4.257	0.861	24.469	1	0.000	0.014
R^2^ = 0.070–0.153/χ^2^ = 71.918 (gl = 6, *p* < 0.000)/% global = 90.7						
**Chronic Allergy**	**B**	**E.T.**	**Wald**	**Gl**	**Sig.**	**Exp(B)**
Age	−0.018	0.009	3.837	1	0.05	0.982
Gender(1)	0.039	0.19	0.041	1	0.839	1.039
Length of residence	0.038	0.014	7.331	1	0.007	1.038
Satisfaction with social support	−0.050	0.13	0.149	1	0.7	0.951
Resilience	0.411	0.192	4.576	1	0.032	1.509
Satisfaction with life	−0.190	0.089	4.492	1	0.034	0.827
Constant	−2.271	0.724	9.837	1	0.002	0.103
R^2^ = 0.014–0.026/χ^2^ = 13.975 (gl = 6, *p* < 0.030)/% global = 86.5						

## Data Availability

The data presented in this study are available on request from the corresponding author.
